# Validation of Active Compound of *Terminalia catappa* L. Extract and Its Anti-Inflammatory and Antioxidant Properties by Regulating Mitochondrial Dysfunction and Cellular Signaling Pathways

**DOI:** 10.4014/jmb.2407.07044

**Published:** 2024-09-02

**Authors:** So Jeong Paik, Dong-Shin Kim, Joe Eun Son, Tran The Bach, Do Van Hai, Jin-Hyub Paik, Sangjin Jo, Dong Joon Kim, Sung Keun Jung

**Affiliations:** 1 School of Food Science and Biotechnology, Kyungpook National University, Daegu 41566, Republic of Korea; 2 National Institute of Horticultural and Herbal Science, Rural Development Administration, Wanju 553635, Republic of Korea; 3 Institute of Ecology and Biological Resources, Vietnam Academy of Science and Technology, 18 Hoang Quoc Viet, Cau Giay, Ha Noi, Vietnam; 4 International Biological Material Research Center, Korea Research Institute of Bioscience & Biotechnology, Daejeon 34141, Republic of Korea; 5 Department of Microbiology, College of Medicine, Dankook University, Cheonan 31116, Republic of Korea; 6 Multidrug-resistant Refractory Cancer Convergence Research Center (MRCRC), Dankook University, Cheonan 31116, Republic of Korea; 7 Research Institute of Tailored Food Technology, Kyungpook National University, Daegu 41566, Republic of Korea

**Keywords:** *Terminalia catappa* L., functional food, inflammation, signaling pathway, mitochondria, ultraperformance liquid chromatography-quadrupole-time-of-flight mass spectrometry

## Abstract

As chronic inflammation and oxidative stress cause various diseases in the human body, this study aimed to develop functional materials to prevent inflammation and oxidative stress. This study investigated the biological function and components of *Terminalia catappa* L. extract prepared using its leaves and branches (TCE). TCE was determined using ultraperformance liquid chromatography-quadrupole-time-of-flight mass spectrometry. Using RAW 264.7 mouse macrophages, inhibitory effects of the identified compounds on nitric oxide (NO) and reactive oxygen species (ROS) generation were analyzed. Therefore, α-punicalagin was selected as an active compound with the highest content (986.6 ± 68.4 μg/g) and physiological activity. TCE exhibited an inhibitory effect on lipopolysaccharide (LPS)-induced inflammatory markers, including NO, inducible nitric oxide synthase, and inflammatory cytokines without exerting cytotoxicity. Moreover, TCE prevented excessive ROS production mediated by LPS and upregulated hemeoxygenase-1 expression via the nuclear translocation of nuclear factor erythroid 2-related factor 2. Interestingly, TCE prevented LPS-induced mitochondrial membrane potential loss, mitochondrial ROS production, and dynamin-related protein 1 phosphorylation (serine 616), a marker of abnormal mitochondrial fission. Furthermore, TCE considerably repressed the activation of LPS-induced mitogen-activated protein kinase pathway. Thus, TCE is a promising anti-inflammatory and antioxidant pharmaceutical or nutraceutical, as demonstrated via mitochondrial dysfunction and cellular signaling pathway regulation.

## Introduction

Inflammation is an essential biological process for maintaining homeostasis in response to several factors, including pathogens, toxic substances, and environmental stress [1, 2]. Inflammatory responses are related to protecting the host [3]; however, excessive inflammatory responses induce the occurrence of chronic inflammation and development of noncommunicable diseases, including chronic respiratory disease, cancer, heart disease, and diabetes [4]. Macrophages are important immune cells in mammals that can recognize invading pathogens and molecules, including lipopolysaccharide (LPS). Macrophages produce excessive nitric oxide (NO) and reactive oxygen species (ROS) for protection from external stimuli; however, this process exerts oxidative stress on host cellular components [5].

Mitochondria are double-membrane organelles that are present in mammalian cells and regulate oxidative stress and immune responses [6]; however, damaged mitochondria can lead to mitochondrial dysfunction, which results in impaired oxidative capacity [7]. The loss of mitochondrial membrane potential (ΔΨ_M_) is associated with biological phenomena causing oxidative stress [8] and induces mitochondrial dysfunction [9]. Overproduction of mitochondrial ROS (mtROS) influences ΔΨ_M_, and subsequent mitochondrial damage can amplify the oxidative stress [10]. In addition, unbalanced mitochondrial fission and fusion cause mitochondrial dysfunction; therefore, it is important to regulate the fusion and fission markers, such as dynamin-related protein 1 (Drp1) [11]. Thus, the maintenance of mitochondrial homeostasis can be a promising strategy for alleviating oxidative stress and inflammation.

The progression of the intracellular signal transduction pathway can cause the advancement of inflammatory diseases [12]. The canonical inflammatory transcription factor nuclear factor-kappa B (NF-κB) transcribes inducible nitric oxide synthase (iNOS) and inflammatory cytokines [13, 14]. Additionally, activated mitogen-activated protein kinases (MAPKs) induce the secretion of inflammatory cytokines by trans-activating activator protein 1 (AP-1) [15]. In addition to inflammatory responses, oxidative stress is dependent on antioxidative cellular enzymes. Hemeoxygenase-1 (HO-1)/nuclear factor erythroid 2-related factor 2 (NRF2) signaling is activated, which responds rapidly to free hemes that exert oxidative stress on cellular components [16]. Thus, modulation of signaling molecules is a promising strategy for preventing inflammation and oxidative stress.

Herbs are traditional plant resources that have long been used in cooking, preservatives, and medicines owing to their physicochemical properties and health benefits [17]. *Terminalia catappa* L. is a natural herb mainly cultivated in tropical areas. Approximately 700,000 tons of this plant are produced annually [18, 19], and its leaves are commonly consumed in the form of tea in traditional societies [20]. In addition, these leaves reportedly possess antimicrobial [21], hepatoprotective [22], antimetastatic [23], and antidiabetic [24] properties, and contain phytochemicals [25] including ellagic acid [26], flavone glycosides, vitexin, and rutin [27]. However, the components and physiological efficacy of *T. catappa* L. extract prepared using its leaves and branches (TCE), a commonly utilized form [24], have not yet been investigated. Therefore, we examined the preventive efficacy of TCE on inflammation and oxidative stress and hypothesized that the cell signaling pathway and organelle homeostasis are related to TCE function.

This study aimed to identify the availability of TCE as a functional material. In addition, we investigated the constituents of TCE to identify bioactive compounds that drive anti-inflammatory and antioxidant properties for food industrial standardization. Furthermore, we sought to elucidate the preventive mechanism of TCE by regulating cell signaling pathways and mitochondrial functional in RAW 264.7 cells.

## Material and Methods

### TCE Preparation

The *T. catappa* L. extract used in this research was collected from the Nghia Do community (Cau Giay district, Vietnam). Dr. Tran The Bach collected and identified the plant samples in June 2010. The acquired voucher specimens (VK 3791) were stored in the herbarium of the Korea Research Institute of Bioscience and Biotechnology (Republic of Korea). *T. catappa* L. leaves and branches (104 g), dried in the shade and powdered, were added to 1 L of 99.9% methyl alcohol (HPLC grade) and extracted through 30 cycles (40 KHz, 1,500 W, 15 min ultrasonication, 120 min standing per cycle) at 20–25°C using an ultrasonic extractor (SDN-900H; SD- Ultrasonic Co., Ltd.). After filtration (Qualitative Filter No. 100; Hyundai Micro Co., Ltd., Republic of Korea) and drying under reduced pressure, the extract (8.61 g) was obtained, with an extraction yield represented as 8.28%. We aliquoted the extract, with each vial containing 20.38 ± 0.13 mg of the extract. The extract was prepared to make a 100 mg/mL concentration by diluting it using dimethyl sulfoxide for *in vitro* experiments.

### Compound Analysis Using Ultraperformance Liquid Chromatography-Quadrupole-Time-of-Flight Mass Spectrometry (UPLC–QTOF MS)

Lyophilized raw materials were pulverized, and 40 mg of the powdered sample was homogenized with 800 μL of 100% methanol using a bullet blender (Next Advance, USA). After centrifugation (14,000 rpm, 10 min, 4°C), 1 μL of clear supernatant was analyzed using an UPLC–QTOF mass spectrometer (Vion; Waters Corp., USA). Punicalin and punicalagin (MedChemExpress, USA), gallic acid (ACROS Organics, Belgium), and ellagic acid (Sigma-Aldrich, USA) were prepared as standards. Punicalin is a mixture of α- and β-punicalin, comprising two-thirds and one-third of α and β configurations, respectively. Additionally, punicalagin is a mixture of 70.68% α-punicalagin and 29.32% of β-punicalagin. An Acquity UPLC BEH C18 column (2.1 × 100 mm, 1.7 μm; Waters Corp.) was equipped with and equilibrated under previously described conditions [28] with slight modifications. Negative electrospray ionization (ESI) mode was utilized to detect the elution of compounds. The following optimized MS conditions were used: desolvation temperature (400°C), desolvation gas flow rate (900 L/h), ion source temperature (100°C), sampling cone voltage (20 V), and capillary voltage (2.5 kV). Leucine–enkephalin ([M–H] = m/z 554.2615) was used as the lock mass. TOF-MS was performed with an m/z range of 100–1,500 and a scan time of 0.2 s. The target compounds were quantified in multiple reaction monitoring (MRM) mode. Each standard mass was injected into the UPLC–QTOF MS system to determine its retention time and fragment ions. The results of precursor ion, product ion, and collision energy are shown in Table 2. UNIFI software (Ver. version 1.9.2, Waters Corp.) was utilized for data acquisition and processing.

### Cell Culture

The mouse macrophage cell line RAW 264.7 (Korean Cell Line Bank, Republic of Korea) was incubated in Dulbecco’s modified Eagle’s medium (DMEM)/high glucose containing 4-mM L-glutamine with 10% fetal bovine serum, and 1% penicillin–streptomycin solution (Thermo Scientific HyClone, USA). The cells were incubated in a humidified incubator (5% CO_2_, 37°C; Eppendorf, Germany).

### NO Production

To detect NO production, RAW 264.7 cells (3 × 10^5^ cells/mL) were grown in 96-well plates for 1 day. TCE (25, 50, and 100 μg/mL) and its compounds (12.5, 25, and 50 μM) were pretreated for 1 h before 1 μg/mL of LPS (LPS25; Sigma-Aldrich) treatment. NO quantification was performed using the acquired medium in which the cells were reacted for 24 h according to previously reported methods [29].

### Western Blot Assay

Lysis buffer (Cell Signaling, USA) supplemented with phosphatase and protease inhibitor (Thermo Fisher Scientific, USA) was used for cell harvesting. Proteins were quantified using a DC protein assay kit (Bio-Rad Inc., USA) and separated via electrophoresis using sodium dodecyl sulfate–polyacrylamide gel. They were transferred to Immobilon polyvinylidene fluoride membranes (0.45 μm; Millipore, USA). After reacting the proteins with 5%skim milk diluted in 1× Tris-buffered saline and 0.1% Tween 20 Detergent, primary antibodies against iNOS, COX-2, KEAP1, HO-1, α/β-tubulin, p-Drp1 S616, Drp1, p-IκBα, IκBα, p-p65, p65, p-SAPK/JNK, SAPK/JNK, p-p38 MAPK, p38 MAPK, p-p44/42 MAPK, p44/42 MAPK, p-c-Jun, c-Jun (Cell Signaling), NRF2, Lamin B1 (Abcam, UK), and β-actin (Santa Cruz Biotechnology, USA) were reacted with membranes (4°C, overnight). The secondary antibodies corresponding to the primary antibodies were reacted with the membranes for 1 h. EzWestLumi plus kit (ATTO, USA) and GeneGnome XPQ NPC system (Syngene, UK) were used to detect and visualize the protein expression. Moreover, the ImageJ software (National Institutes of Health, USA) was employed to calculate the exact expression level.

### Evaluation of Intracellular ROS Feneration

To determine the ROS level, TCE and single compounds were reacted with the cells. After 1 h, LPS was added, and 24 h later, 20 μM of 2',7'-dichlorofluorescein diacetate (DCF-DA) solution was reacted with the cells for 30 min. Fluorescent plate reader (485–538 nm; Molecular Devices Corp., USA) and fluorescence microscopy (Leica Microsystems, Germany) were used for ROS measurement and observation.

### Evaluation of mRNA Expression Using Quantitative Real-Time Polymerase Chain Reaction (qRT–PCR)

TCE was reacted with the cells, and LPS was added 1 h later. For total RNA extraction, RNAIso Plus (TAKARA, Japan) was used. Genomic DNA (gDNA) remover (TOYOBO, Japan) was used to eliminate DNA contaminant, and SYBR Green Realtime PCR Master Mix (TOYOBO) was used for qRT–PCR runs. The relative mRNA levels were calculated using the comparative ΔΔCq method. The nucleic acid sequences of the primers (interleukin[IL]-6, IL-1β, and GAPDH) are presented in Table 1.

### Cytotoxicity Test

After reacting the cells with TCE (24 h), surviving cell rate was measured following a previously described method [29].

### Cytosol and Nuclear Fraction

The cells were reacted with TCE and subjected to LPS treatment after 1 h. After incubation, protein separation was performed using NE-PER Nuclear and Cytoplasmic Extraction Reagents containing protease and phosphatase inhibitor (Thermo Fisher Scientific) according to the manufacturer’s protocol.

### Measurement of Free Radical Scavenging Capacity

Using previously reported methods, the effect of the samples on free radicals was evaluated with slight modifications [29]. Briefly, 2,2’-Azino-bis(3-ethylbenzothiazoline-6-sulfonic acid) (ABTS) scavenging activity was evaluated using 5 mg/mL of ABTS tablet (Sigma-Aldrich) with potassium persulfate (2.45 mM) dissolved in distilled water and subsequently diluted with sterilized phosphate-buffered saline. Subsequently, 100 μL/well of TCE (25, 50, and 100 μg/mL) and ascorbic acid (0.125, 0.25, 0.5, 1, 2, and 4 μg/mL) were transferred to 96-well plates, and the same amount of diluted ABTS solution was added. At a 750-nm wavelength, the absorbance was detected using a microplate reader (Molecular Devices Corp.,) following the reaction for 30 min. 2,2-Diphenyl-1-(2,4,6-trinitrophenyl)hydrazin-1-yl (DPPH) radical scavenging activity was assessed using a DPPH solution (40 μg/mL) diluted in methanol, and TCE (25, 50, and 100 μg/mL) and ascorbic acid (0.25, 0.5, 1, 2, 4, and 8 μg/mL) diluted in methanol were transferred to 96-well plates at 100 μl/well. The DPPH solution was supplemented with the same amounts of samples. At a 595-nm wavelength, the absorbance was detected using a device similar to that of ABTS.

### Evaluation of Mitochondrial Membrane Potential

Cells were grown in eight-well chambers (Ibidi, Germany) and incubated overnight. To evaluate the time dynamics of ΔΨ_M_, LPS was reacted with the cells for 0.5, 1, and 3 h, respectively. To evaluate the effect of TCE on ΔΨ_M_ loss, TCE was reacted with the cells for 1 h, and subsequently LPS was reacted for 3 h. JC-1 (1 μg/mL) dye (Molecular Probes, USA) was incubated with the cells for 15 min. Nuclear staining was performed using mounting media (Abcam), and JC-1 aggregates and monomers were visualized using a fluorescence microscope (Leica Microsystems).

### Evaluation of mtROS Production

Cells were grown in eight-well chambers and incubated overnight. To determine the effect of TCE on mitochondrial superoxide content, TCE (50 and 100 μg/mL) was treated with the cells, and then LPS was added for 1 h. Live cell imaging was performed by incubating cells with MitoSOX Red mitochondrial superoxide indicator (5 μM; Molecular Probes) for 10 min. Fixation with 4% paraformaldehyde was performed to stop the reaction within the cells, and a mounting medium (Abcam) was used for nuclear staining. mtROS was observed using a fluorescence microscope (Leica Microsystems).

### Immunofluorescence

The cells were seeded into eight-well chambers (Ibidi), and TCE was added to the cells before LPS treatment. After 30 min of reacting the cells with LPS, the cells were incubated with 4% formaldehyde, after which 100%methanol stored in a deep freezer was used for cell permeabilization. C-Jun antibody was reacted with the cells at 4°C overnight, and then goat anti-rabbit IgG H&L conjugated to Alex Fluor 488-conjugated antibodies were added. A fluorescence microscope (Leica Microsystems) was used for assessment of c-Jun expression.

### Statistical Analysis

Experimental values were calculated as mean ± standard deviation or standard error. Each experiment was repeated at least thrice for its accuracy. A *P* < 0.05 was used to indicate statistical significance and measured with one-way ANOVA and Dunnett’s *post-hoc* test using the GraphPad Prism (GraphPad; USA).

## Results

### Targeted Identification and Quantification Analysis of TCE Compounds Using UPLC–QTOF MS

**Fig. 1:**
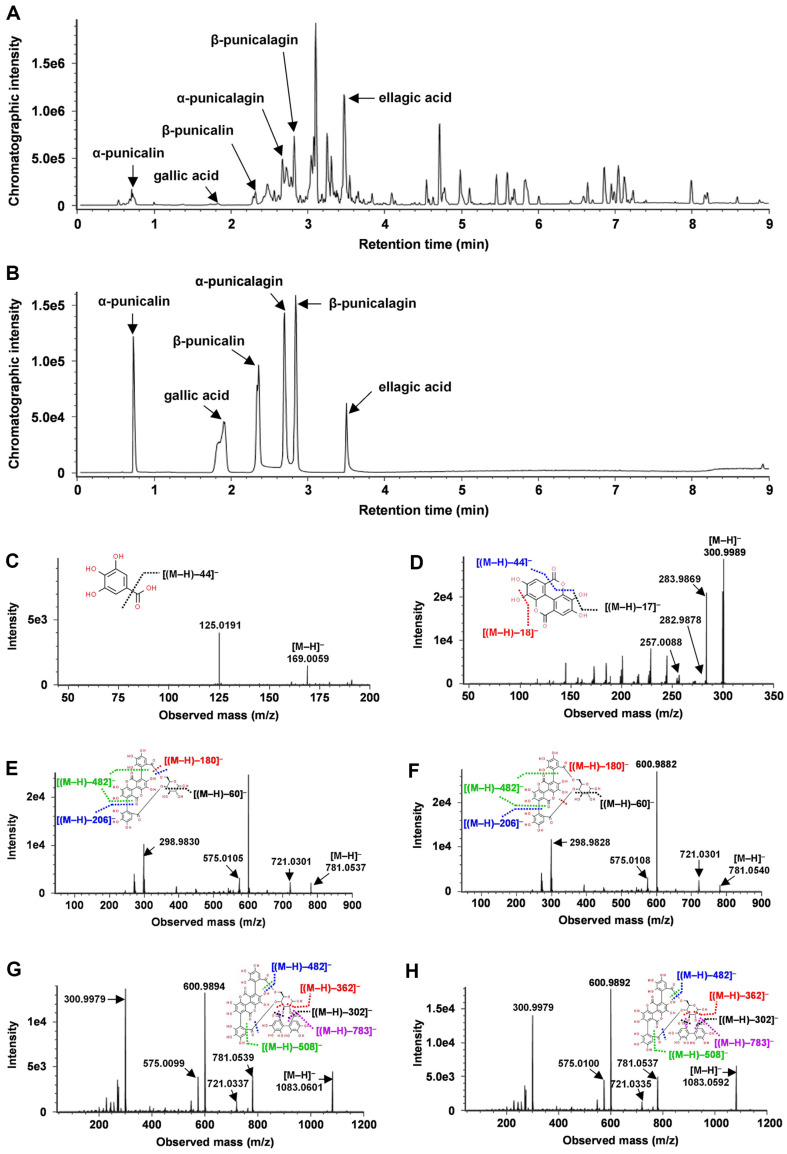
Representative chromatograms and mass spectrometry (**MS**)/MS spectra of the targeted phenolic compounds analyzed using ultraperformance liquid chromatography-quadrupole-time-of-flight mass spectrometry (UPLC–QTOF MS). The chromatograms of (**A**) extract of *T. catappa* L. leaves and branches and (**B**) standard chemicals. The MS/MS spectra of (**C**) gallic acid, (**D**) ellagic acid, (**E**) α-punicalin, (**F**) β-punicalin, (**G**) α-punicalagin, and (**H**) β-punicalagin were analyzed using quantitative UPLC–QTOF MS/MS with a negative ESI mode. MS/MS spectra were obtained using collision energies of 10–30, 30–50, or 50–70 eV.

In the present study, >100 botanical extracts were screened to develop anti-inflammatory materials for the prevention of inflammatory disease. Among the screened samples, the top five materials with the highest NO inhibitory properties were *Terminalia catappa* L., *Castanopsis fissa* (Champ. ex Benth.) Rehder & E.H. Wilson, *Rhaphiolepis indica* (L.) Lindl., *Lindera chunii* Merr., and *Macrosolen cochinchinensis* (Lour.) Tiegh., and TCE was selected as a candidate material with the highest NO inhibitory effect (data not shown). To develop botanical materials as functional food and pharmaceutical materials, it is important to use indicators or active compounds. Therefore, we selected compounds through literature analysis and qualitative and quantitative analyses were performed using UPLC–QTOF MS/MS. A comparison between the UPLC chromatograms of standard chemicals and TCE showed that TCE contains α-punicalin, β-punicalin, α-punicalagin, β-punicalagin, gallic acid and ellagic acid (Fig. 1A and 1B). Gallic acid ([M‒H]‒ = m/z 169.0069) produced fragment ions at m/z 125 (Fig. 1C), and ellagic acid ([M‒H]‒ = m/z 300.9989) produced fragment ions at m/z 283, 282, and 257 (Fig. 1D). Punicalins (α-:[M‒H]‒ = 781.0537 and β-: [M‒H]‒ = 781.0540) and punicalagins (α-: [M‒H]‒ = 1,083.0601 and β-: [M‒H]‒ = 1,083.0592) produced the same fragment ions at m/z 600, 298, 575, and 721 and m/z 600, 300, 781, 575, and 721 (Fig. 1E–1H).

Based on the fragmentation patterns, the targeted phenolic compounds present in TCE were quantified using the MRM mode, and the contents are displayed in Table 2. Among the phenolic compounds, α-punicalagin showed the highest content (986.6 ± 68.4 μg/g dry sample), followed by α-punicalin (385.7 ± 26.6 μg/g dry sample), ellagic acid (320.1 ± 19.4 μg/g dry sample), β-punicalagin (304.9 ± 21.1 μg/g dry sample), and β-punicalin (152.0 ± 4.4 μg/g dry sample). Among the targeted compounds, gallic acid showed the lowest content (11.1 ± 1.4 μg/g dry sample).

### Identification of Active Compounds of TCE with Anti-Inflammatory and Antioxidant Properties in RAW 264.7 Cells

**Fig. 2:**
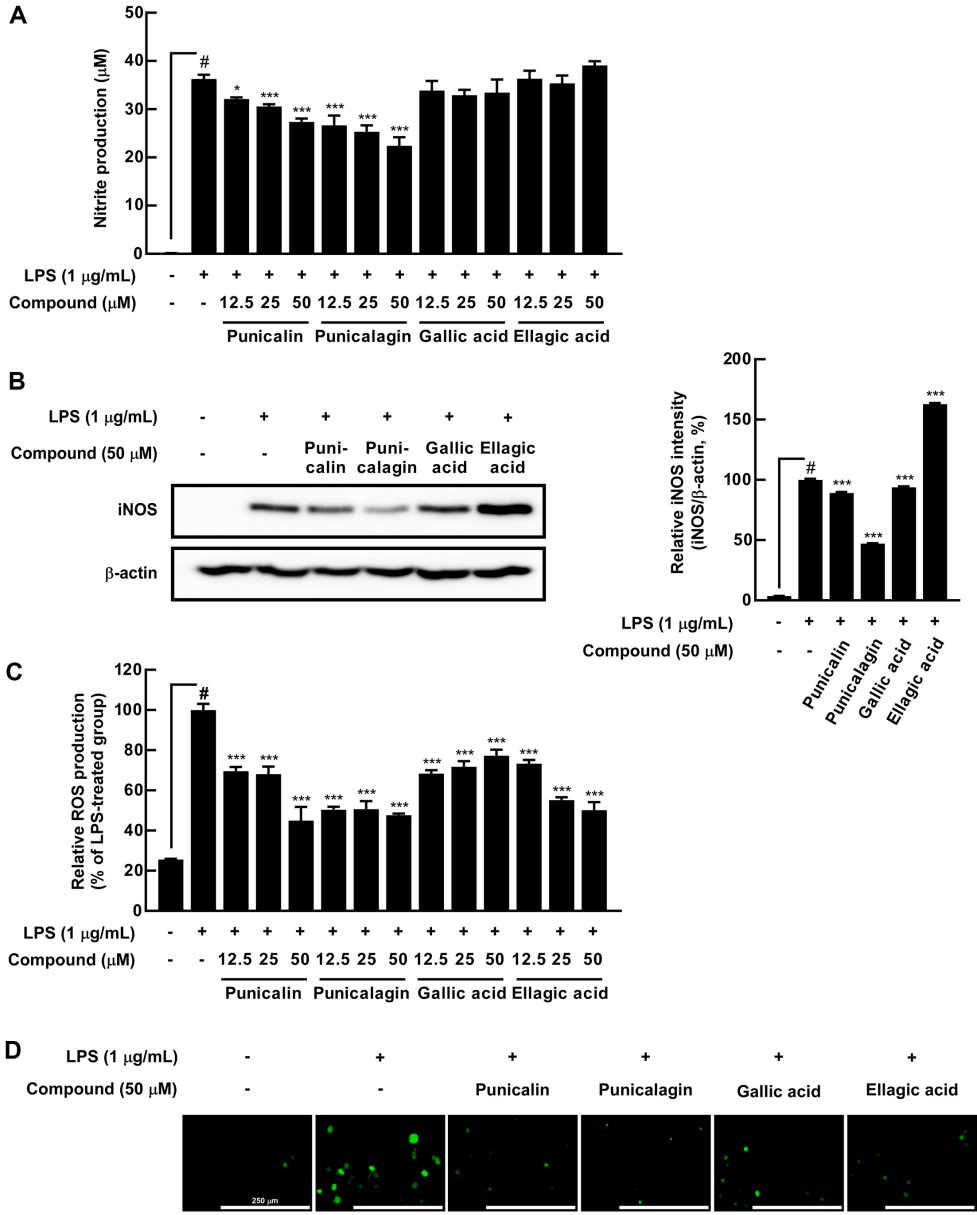
Effects of single compounds present in *Terminalia catappa* L. extract prepared using leaves and branches (**TCE**) on the lipopolysaccharide (**LPS**)-induced nitric oxide (**NO**) production, inducible nitric oxide synthase (iNOS) expression, and reactive oxygen species (**ROS**) production in RAW 264.7 cells. (**A**) NO production was evaluated using the Griess assay. (**B**) iNOS expression was visualized using western blot assay, and the relative iNOS band intensity was quantified using ImageJ. (**C**) ROS production was measured using 2',7'-dichlorofluorescein diacetate (DCF-DA), and (**D**) DCF-DA localization was visualized using fluorescent microscopy; 20× objective, scale bar = 250 μm. Cells are pretreated with the compounds (50 μM) for 1 h and then treated with LPS (1 μg/mL). # *p* < 0.05, compared with the control group; * *p* < 0.05 and *** *p* < 0.001, compared with the only LPS-treated group. Values are expressed as means ± standard deviations (SDs) of three individual experiments.

To verify the active compounds of TCE, the inhibitory effects of TCE compounds on NO and ROS generation caused by LPS were compared as inflammation and oxidative stress markers, respectively. Among all TCE compounds, punicalin and punicalagin markedly suppressed LPS-induced NO production; however, gallic acid and ellagic acid showed no such effect. Compared with the LPS-treated group, punicalagin showed the highest NO inhibitory effect at 26.5%, 30.2%, and 38.3% (25, 50, and 100 μg/mL), whereas punicalin inhibited NO production by 11.5%, 15.7%, and 24.6% (Fig. 2A). Still, compounds did not exhibit cytotoxicity (Fig. S1). LPS-induced iNOS expression was markedly suppressed by punicalagin, followed by punicalin; however, gallic acid and ellagic acid showed no such effect. iNOS band intensity quantification showed that punicalagin inhibited iNOS expression by 53% compared with the LPS-treated group, whereas punicalin had a 10.9% inhibitory effect (Fig. 2B). Using DCF-DA as the ROS probe, we confirmed that all compounds significantly inhibited ROS production. Punicalagin showed 49.6%, 49.4%, and 52.3% lower ROS production compared with the LPS-treated group (25, 50, and 100 μg/mL), whereas punicalin exhibited 30.5%. 31.9%, and 55.1% lower ROS production. Ellagic acid showed 26.7%, 44.8%, and 49.8% lower ROS production, followed by gallic acid at 31.7%, 28.3%, and 22.8% (Fig. 2C and 2D). Therefore, we determined punicalagin as an active compound of TCE with anti-inflammatory and antioxidant activities.

### TCE Suppressed NO Production, iNOS Expression, and Proinflammatory Cytokine Expression Mediated by LPS in RAW 264.7 Cells

**Fig. 3:**
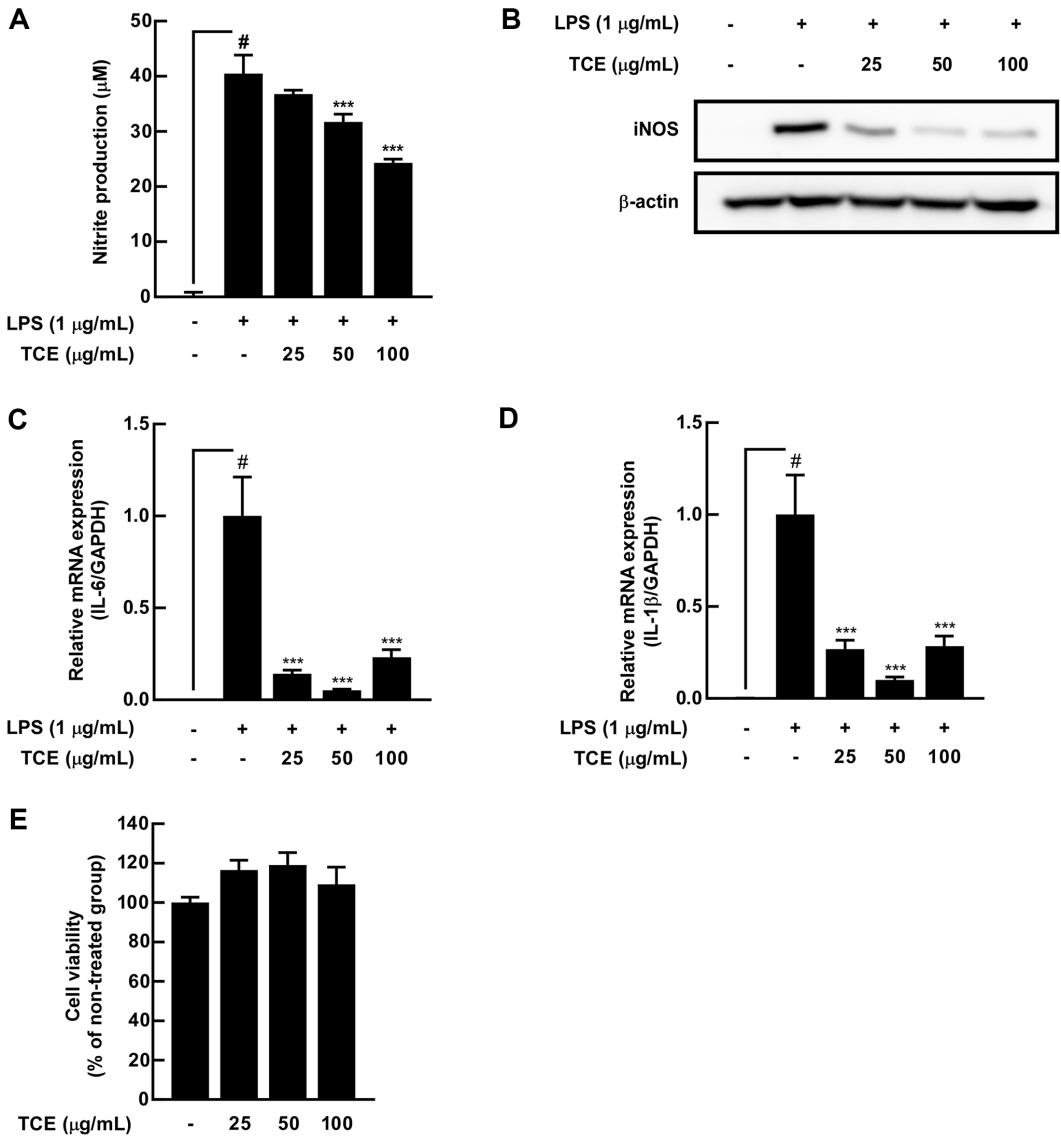
Effects of TCE on LPS-induced NO production, iNOS expression, proinflammatory cytokine expression, and viability in RAW 264.7 cells. (**A**) NO production was evaluated using Griess assay. (**B**) iNOS expression was visualized using western blot assay. The mRNA expression of (**C**) interleukin (**IL**)-6 and (**D**) IL-1β was measured using quantitative real-time polymerase chain reaction. Relative mRNA expression is represented by the normalization of each target gene expression based on GAPDH expression. (**E**) Cell viability was evaluated using MTT assay. # *p* < 0.05, compared with the control group; *** *p* < 0.001, compared with the only LPS-treated group. Values are expressed as means ± SDs or mean ± standard error of mean (**SEM**) of three individual experiments.

We confirmed that punicalagin is an active TCE compound and further evaluated its ability to inhibit LPS-induced NO production in RAW 264.7 cells. TCE significantly inhibited LPS-induced NO production (Fig. 3A). iNOS can elevate NO production [30], and we observed that TCE inhibited the LPS-induced iNOS expression (Fig. 3B). Inflammation can be induced by the secretion of proinflammatory cytokines, including IL-1β and IL-6 [31], and we examined whether TCE inhibited the mRNA expression of proinflammatory cytokines mediated by LPS. TCE inhibited the expressions of IL-6 and IL-1β (Fig. 3C and 3D). In addition, TCE did not exert any cytotoxic effects (Fig. 3E).

**Fig. 4:**
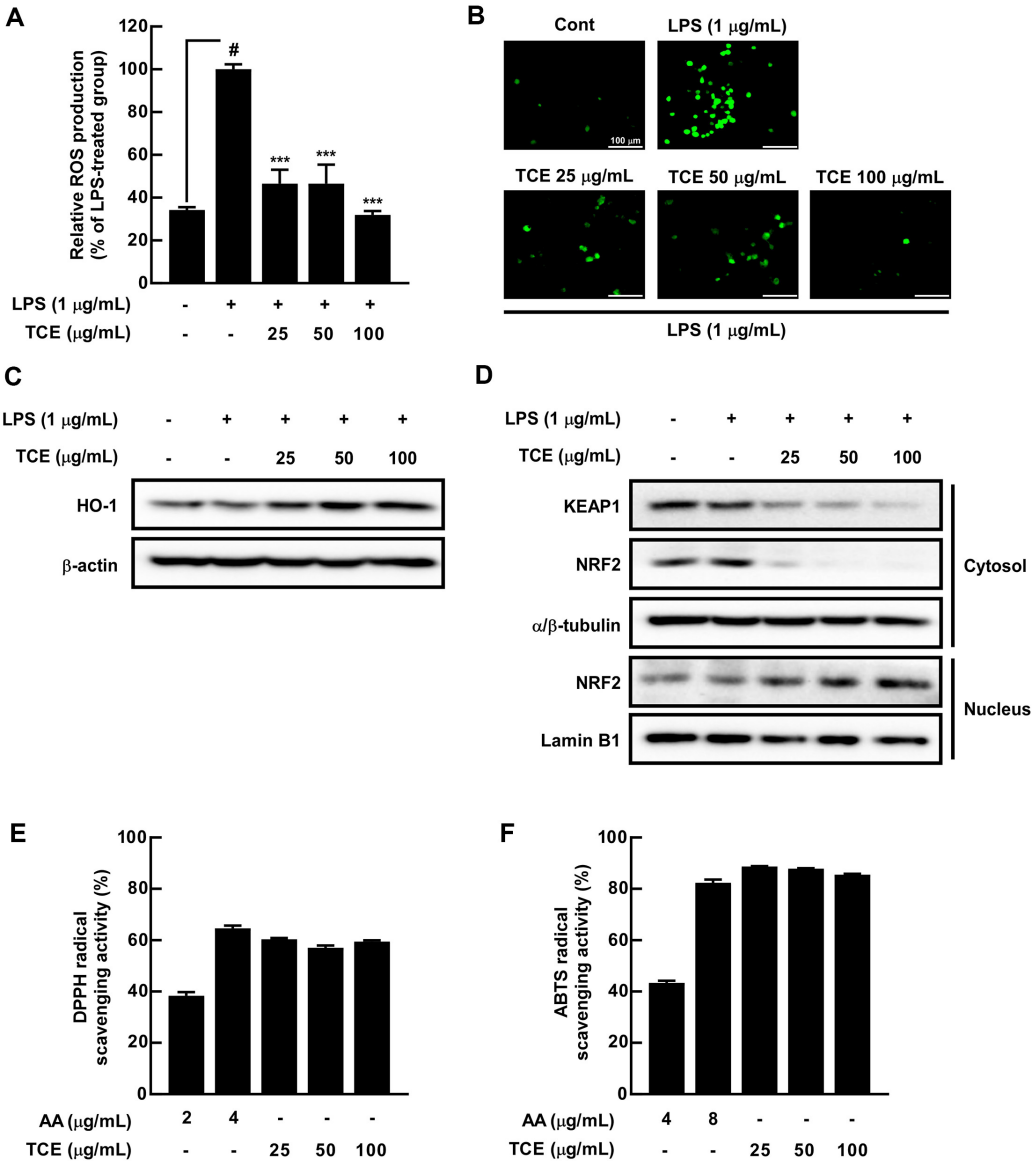
Effects of TCE on LPS-induced ROS production via hemeoxygenase-1 (HO-1)/nuclear factor erythroid 2-related factor 2 (NRF2) signaling pathway activation in RAW 264.7 cells. (**A**) ROS production was measured using DCF-DA, and (**B**) DCF-DA localization was visualized using a fluorescent microscope; 40× objective, scale bar = 100 μm. (**C**) HO-1 expression was detected using western blot assay. (**D**) KEAP1 and NRF2 expression in the cytosol and nucleus were detected using western blot after cytosol/nuclear fractionation. The free radical scavenging effect was measured using (**E**) 2,2-Diphenyl-1-(2,4,6-trinitrophenyl)hydrazin-1-yl (**DPPH**) and (**F**) 2’-Azino-bis(3-ethylbenzothiazoline-6- sulfonic acid) (**ABTS**) radical scavenging activity assays. # *p* < 0.05, compared with the control group; *** *p* < 0.001, compared with the only LPS-treated group. Values are expressed as means ± SDs of three individual experiments.

TCE Suppressed LPS-Induced ROS Production by Activating HO-1/NRF2 Signaling Pathway in RAW 264.7 Cells The occurrence of inflammation is associated with ROS in terms of their inflammatory reactions and oxidative stress [32]. TCE significantly inhibited ROS production mediated by LPS in RAW 264.7 cells (Fig. 4A and 4B). HO-1 affects the cell system, and its activation regulates ROS generation [33]. We investigated whether TCE activates HO-1 expression and observed that TCE treatment increased HO-1 expression (Fig. 4C). HO-1 is a downstream factor of NRF2 [34], and the nuclear translocation of NRF2 results in KEAP1 inactivation [35]. TCE downregulated KEAP1 expression in a dose-dependent manner, and NRF2 expression was constant in whole cell lysates (Fig. S2). TCE downregulated KEAP1 expression in a dose-dependent manner, and NRF2 expression was reduced in the cytoplasm. In addition, NRF2 expression in the nucleus was increased by TCE treatment in a dose-dependent manner (Fig. 4D). Furthermore, we established that TCE can scavenge DPPH and ABTS free radicals (Fig. 4E and 4F).

### TCE Prevented LPS-Induced Mitochondrial Dysfunction in RAW 264.7 Cells

**Fig. 5:**
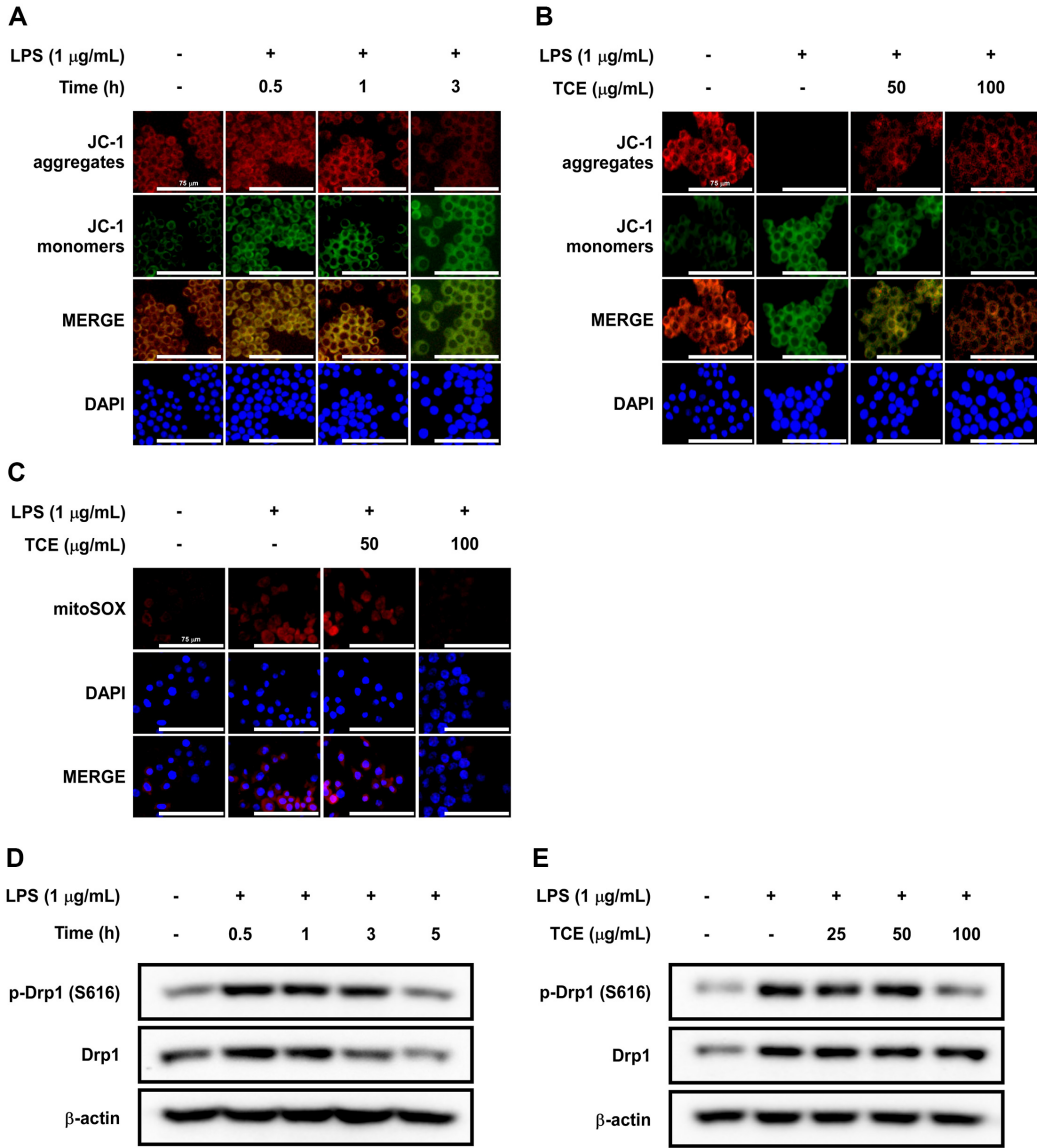
Effects of TCE on LPS-induced mitochondrial dysfunction in RAW 264.7 cells. (**A**) The time dynamics of ΔΨ_M_ were visualized via fluorescent microscopy using JC-1 dye; 20× objective, scale bar = 75 μm. (**B**) ΔΨ_M_ was visualized via fluorescence microscopy using JC-1 dye; 20× objective, scale bar = 75 μm. Cells were pretreated with TCE for 1 h before LPS treatment and incubated for 3 h following LPS treatment. (**C**) mtROS were visualized via fluorescence microscopy using MitoSOX^TM^ Red reagent; 20× objective, scale bar = 75 μm. Cells were pretreated with TCE for 1 h and incubated for 3 h following LPS treatment. (**D**) The time dynamics of p-Drp1 (S616) and Drp1 expression for LPS treatment time were detected using western blot assay. (**E**) p-Drp1 and Drp1 expression were detected using western blot assay. Cells were pretreated with TCE for 1 h and then incubated for 30 min following LPS treatment.

Loss of mitochondrial function can lead to oxidative stress via the excessive generation of mtROS [36] and inflammation owing to the secretion of proinflammatory cytokines [37]. We evaluated ΔΨ_M_, a marker of mitochondrial dysfunction, using the JC-1 dye. The levels of LPS-induced JC-1 aggregates decreased over time, whereas the levels of JC-1 monomers increased. Incubation for 3 h after LPS treatment was selected as the appropriate condition for measuring ΔΨ_M_ (Fig. 5A). Although TCE significantly decreased the levels of JC-1 monomers, it increased the levels of JC-1 aggregates in RAW 264.7 cells (Fig. 5B). ΔΨ_M_ is affected by excessive mtROS production [10], and we investigated the ability of TCE to inhibit LPS-induced mtROS production. The results indicated that TCE significantly suppressed mtROS production caused by LPS (Fig. 5C). As Drp1 is linked to mitochondrial fission [38], we examined the time dynamics of Drp1 response to LPS treatment. LPS treatment enhanced Drp1 S616 phosphorylation 30 min after treatment and then decreased it over time (Fig. 5D). Hence, TCE markedly suppressed LPS-induced Drp1 phosphorylation (Fig. 5E).

### TCE Suppressed LPS-Induced JNK/c-Jun and p38 Phosphorylation and c-Jun Expression in the Cytosol and Nucleus in RAW 264.7 Cells

**Fig. 6:**
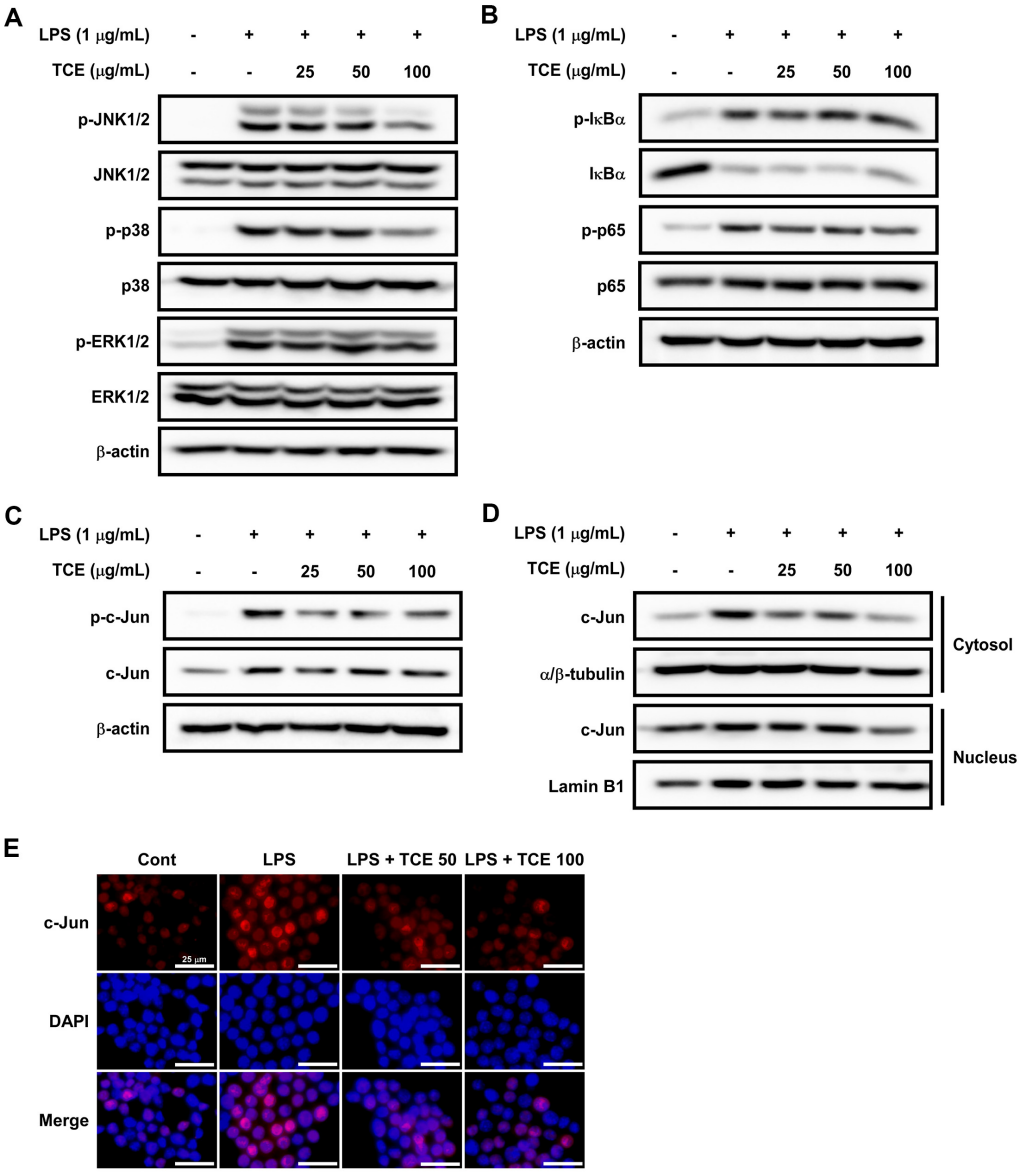
Effects of TCE on LPS-induced mitogen-activated protein kinases (**MAPK**) and nuclear factor-kappa B (NF-κB) signaling pathways, and c-Jun expression in RAW 264.7 cells. (**A**) Expression of MAPK and (**B**) NF-κB signaling pathways, (**C**) c-Jun in whole cell lysates were detected using western blot assay. (**D**) c-Jun expression in the cytosol and nucleus was detected using western blot assay following cytosol/nucleus fractionation, and (**E**) c-Jun localization was visualized using fluorescence microscopy based on immunofluorescence; 20× objective, scale bar = 25 μm.

iNOS-induced NO levels and gene expression of cytokines can be regulated by cellular signaling pathways such as MAPK [39] and NF-κB [40]. Therefore, we explored whether TCE affects MAPK and NF-κB signaling cascades in RAW 264.7 cells. The findings indicated that TCE inhibited JNK1/2 and p38 phosphorylation mediated by LPS; however, ERK1/2 was not inhibited (Fig. 6A). Interestingly, TCE did not significantly affect IκBα and p65 phosphorylation induced by LPS (Fig. 6B). A downstream kinase of JNK, c-Jun activation induces AP-1 formation, leading to inflammation [41]. TCE significantly suppressed c-Jun phosphorylation and expression induced by LPS (Fig. 6C). In addition, we proved that TCE inhibited c-Jun expression in the cytoplasm and nucleus (Fig. 6D and 6E).

## Discussion

Cancer, chronic respiratory disease, diabetes, and heart disease are chronic inflammatory diseases, and medication or treatment is immediately required to cure these conditions; however, preventing fatal noninfectious diseases by alleviating inflammation is a better strategy than curing them in terms of treatment time, cost, and patient and family suffering. We aimed to develop anti-inflammatory materials for prevention and selected TCE as a candidate material with the highest NO inhibitory efficacy among the tested botanical extracts. Standardization of raw materials is an important aspect in the functional food industry. To standardize plant-derived functional raw materials, identifying a representative single ingredient, determining the concentration that exhibits biological activity, and maintaining a constant concentration are important processes. We identified punicalin, punicalagin, ellagic acid, and gallic acid as compounds of TCE, and α-punicalagin (986.6 ± 68.4 μg/g) showed high content compared with other compounds. Yakubu *et al*. reported that the highest peak in the LC–MS ion chromatogram of the *T. catappa* leaf was corilagin, and the amount of punicalagin was approximately fivefold lower than that of corilagin [42]. In addition, the ethanolic extract of *T. catappa* L. stem bark contained the highest amount of ellagic acid (6.55 μg/mg extract), followed by the castalagin isomers V (3.45 μg/mg) and IV (2.01 μg/mg), and gallic acid (1.72 μg/mg) [43]. We hypothesized that these differences occurred because of the distinct plant parts used, including branches, stems, and leaves of the plant, although they are of the same species. As most studies have demonstrated that *T. catappa* L. leaves have biological functions and constituents [21-23], our results revealed the potential of *T. catappa* L. branches as a new functional material.

Physiologically active components were explored among the identified compounds, and punicalagin showed the highest anti-inflammatory and antioxidant properties by suppressing NO production and iNOS expression as well as ROS generation. Therefore, we designated punicalagin as the active ingredient of TCE and subsequently evaluated the anti-inflammatory activity of TCE, which contains punicalagin as an active ingredient. Plant extracts can prevent inflammatory diseases by inhibiting excessive NO and cytokine production [44-47], and TCE showed significant inhibitory effect on NO production, iNOS expression, and IL-1β and IL-6 gene expression mediated by LPS in RAW 264.7 cells. These results showed that TCE exerts potent anti-inflammatory effects via inhibiting abnormal NO, IL-6, and IL-1β production. As the experimental concentration (maximum 50 μM) did not match the actual content of punicalagin in TCE (986.6 ± 68.4 μg/g), it is likely that the effect observed by TCE was not solely due to the punicalagin.

ROS exert oxidative stress on intracellular components, thereby resulting in the outbreak of inflammation [48]. To prevent oxidative stress-induced inflammatory diseases, More *et al*. and Hwang *et al*. elucidated the role of plant extracts in inhibiting immoderate ROS production [49, 50]. Using a DCF-DA probe, we confirmed that TCE significantly inhibited LPS-induced ROS generation. Regulation of HO-1/NRF2 signaling pathway is an oxidative stress prevention strategy [51, 52]. TCE upregulated HO-1 expression and downregulated KEAP1 expression in the whole cell; however, NRF2 expression was constant regardless of TCE treatment. We observed that TCE downregulated KEAP1 and NRF2 expression in the cytosol, increasing the nuclear translocation of NRF2. Furthermore, TCE removed free radicals, including DPPH and ABTS, similar to ascorbic acid, a well-known antioxidant [53]. Overall, TCE can be presented as an antioxidant via the regulation of HO-1/NRF2 signaling pathway as well as the removal of free radicals.

Damaged mitochondrial constituents secreted into the cytosol and act as act as damage-associated molecular patterns, causing inflammation [54]. The mitochondrion produces intracellular ROS, and external stimuli-induced mitochondrial dysfunction causes oxidative stress, thereby leading to inflammation [55]. We confirmed that LPS induced ΔΨ_M_ damage, and TCE strongly suppressed the influence in ΔΨ_M_ caused by LPS. Driving ATP production by electron transport and a proton gradient is crucial in mitochondrial respiration; mtROS are byproducts of this process and can cause inflammation-related metabolic diseases [56], but TCE markedly suppressed mtROS overproduction. The phosphorylation of Drp1 residue Ser616 induces the transport of Drp1 to the mitochondrial outer membrane, resulting in mitochondrial dysfunction [57]. We observed that Drp1 phosphorylation was the highest at 30 min after LPS treatment, and it disappeared with decreased expression of Drp1 whole form at 1 h of LPS treatment. As mitochondrial fission involves the decomposition of Drp1 attached to the mitochondrial outer membrane [58], we evaluated whether TCE pretreatment can prevent LPS-induced mitochondrial fission following 30-min LPS treatment, wherein the fission process was considered the most active. We confirmed a distinct inhibitory effect of TCE on the LPS-treated mitochondria fission, and our results showed that TCE can act as a mitochondrial dysfunction regulator against oxidative stress and inflammatory responses.

iNOS expression is regulated by signal transduction pathways, including NF-κB and MAPK [59]. We investigated whether TCE affects NF-κB signaling cascade induced by LPS to identify the preventive mechanism against inflammation. Several studies have reported that natural substances exert inhibitory effects on cellular NF-κB signal transduction pathways [60, 61]; however, TCE showed no effect on LPS-induced IκBα phosphorylation. Therefore, we concluded that the effect of TCE on overall NF-κB signaling molecules was less significant than the inhibitory effect on iNOS expression. We considered the MAPK signaling pathway as another major inflammatory signaling and noted that TCE significantly suppressed JNK1/2 and p38 phosphorylation. Furthermore, protein band quantification revealed that JNK1/2 phosphorylation was the most inhibited among the MAPK signaling molecules (data not shown). JNK is an enzyme that mediates signal transduction by adding phosphate to c-Jun, an element of transcription factor AP-1 [62]. TCE showed inhibitory effect on LPS-induced c-Jun phosphorylation; however, c-Jun expression was also unexpectedly decreased. The downregulation of c-Jun expression resulted in the reduction of inflammatory responses [63, 64], and we confirmed that c-Jun expression was reduced in both cytosol and nucleus; the same effect was observed in intracellular fluorescence analysis. These results showed that TCE suppresses c-Jun downregulation and also induces c-Jun phosphorylation. Therefore, we determined that TCE could prevent the activation of transcription factors associated with inflammatory genes by inhibiting c-Jun phosphorylation and expression.

Compound analysis and *in vitro* assay results showed that TCE, which contains several compounds, including punicalagin, exerts outstanding anti-inflammatory and antioxidant effects. Our study suggested that TCE can become a functional food material using punicalagin as a functional indicator.

## Conclusion

We identified and quantified TCE compounds, which revealed that they contained the highest concentration of punicalagin, followed by punicalin, ellagic acid, and gallic acid. Compared to their inhibitory effect on inflammatory and oxidative markers, we selected punicalagin with the highest efficacy. In addition, we proved that TCE exhibited anti-inflammatory and antioxidant effects by preventing mitochondrial dysfunction, inhibiting the MAPK signaling pathway, and upregulating the HO-1/NRF2 signaling pathway. In conclusion, TCE can be a promising anti-inflammatory and antioxidant material via mitochondrial homeostasis and cellular signaling pathway regulation.

## Supplemental Materials

Supplementary data for this paper are available on-line only at http://jmb.or.kr.



## Figures and Tables

**Table 1:** Nucleic acid sequences of the primers.

Target gene	Sense strand (5'→3')	Antisense strand (3'→5')
GAPDH	AAC TTT GGC ATT GTG GAA GG	ACA CAT TGG GGG TAG GAA CA
IL-1β	GTT GAT GTG CTG CTG CGA GA	AGT TGA CGG ACC CCA AAA GAT
IL-6	AGC CTC CGA CTT GTG AAG TGG T	TGG GAC TGA TGC TGG TGA CAA C

**Table 2:** MRM analysis conditions and quantification of targeted phenolic compounds in TCE.

Compounds	Precursor ion (m/z)	Product ion (m/z)	Collision energy (eV)	Content (μg/g dry sample)
Gallic acid	169.0147	125.0191	20	11.1 ± 1.4
Ellagic acid	300.9964	270.9873	30	320.1 ± 19.4
α-punicalin	781.0537	600.9914	45	385.7 ± 26.6
β-punicalin	781.0540	600.9882	45	152.0 ± 4.4
α-punicalagin	1083.0601	300.9986	45	986.6 ± 68.4
β-punicalagin	1083.0592	300.9986	45	304.9 ± 21.1
