# Supplementary material for: Validation of Active Compound of Terminalia catappa L. Extract and Its Anti-Inflammatory and Antioxidant Properties by Regulating Mitochondrial Dysfunction and Cellular Signaling Pathways
Source: J Microbiol Biotechnol. 2024 Sep 2;34(10):2118–31. doi: 10.4014/jmb.2407.07044 (PMC11540620; doi:10.4014/jmb.2407.07044)
Supplement: Supplementary file 1 [file jmb-34-10-2118-supple.pdf]

J. Microbiol. Biotechnol. 2024. 34(10): 2118-2131

https://doi.org/10.4014/jmb.2407.07044

Supplementary Figures

Validation of Active Compound of *Terminalia catappa* L. Extract and its Anti-inflammatory and Antioxidant Properties by Regulating Mitochondrial Dysfunction and Cellular Signaling Pathways

So Jeong Paik[1], Dong-Shin Kim[2], Joe Eun Son[1], Tran The Bach[3], Do Van Hai[3], Jin-Hyub Paik[4], Sangjin Jo[4], Dong Joon Kim[5,6,*], and Sung Keun Jung[1,7,*]

[1] School of Food Science and Biotechnology, Kyungpook National University, Daegu 41566, Republic of Korea

[2] National Institute of Horticultural and Herbal Science, Rural Development Administration, Wanju 553635, Republic of Korea

[3] Institute of Ecology and Biological Resources, Vietnam Academy of Science and Technology, 18 Hoang Quoc Viet, Cau Giay, Ha Noi, Vietnam

[4] International Biological Material Research Center, Korea Research Institute of Bioscience & Biotechnology, 125, Gwahak-ro, Yuseong-gu, Daejeon 34141, Republic of Korea

[5] Department of Microbiology, College of Medicine, Dankook University, Cheonan, Chungcheongnam-do, 31116, Republic of Korea

[6] Multidrug-resistant Refractory Cancer Convergence Research Center (MRCRC), Dankook University, Cheonan, Chungcheongnam-do, 31116, Republic of Korea

[7] Research Institute of Tailored Food Technology, Kyungpook National University, Daegu 41566, Republic of Korea

[*] Correspondence: djkim407@dankook.ac.kr,
skjung04@knu.ac.kr; Tel.: +82-53-950-7764

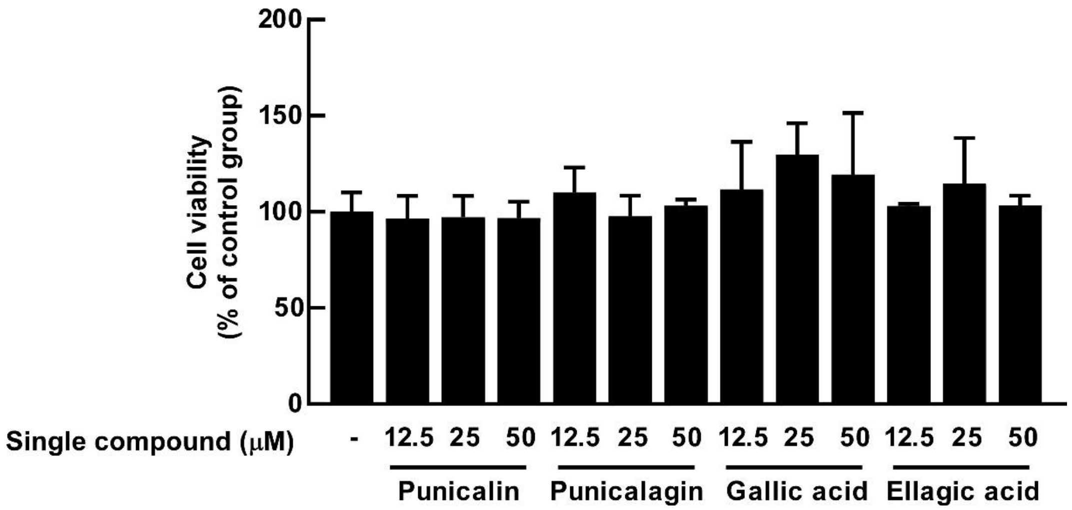

Fig S1. Effects of single compounds present in *Terminalia catappa* L. extract prepared using leaves and branches (TCE) on cell viability in RAW 264.7 cells. Cell viability was evaluated using MTT assay. Values are expressed as means ± SDs of three individual experiments.

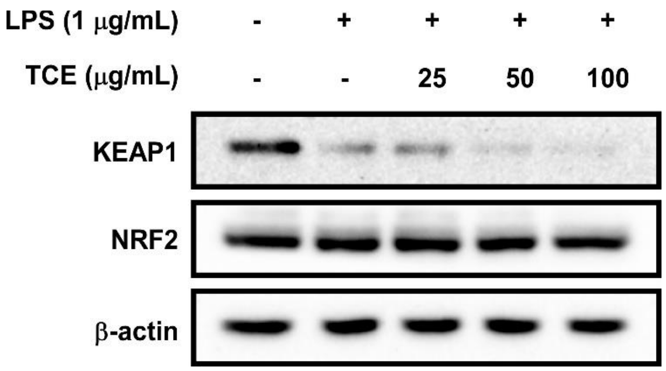

Fig S2. Effects of TCE on LPS-induced hemeoxygenase-1 (HO-1) and nuclear factor erythroid 2-related factor 2 (NRF2) expression in RAW 264.7 cells. KEAP1 and NRF2 expression were detected using western blot assay.
